# Antioxidant and Antiinflammatory Activity of *Vitex negundo*

**DOI:** 10.4103/0250-474X.49140

**Published:** 2008

**Authors:** R. R. Kulkarni, A. D. Virkar, Priscilla D'mello

**Affiliations:** Department of Pharmacognosy and Phytochemistry, Principal K.M. Kundnani College of Pharmacy, Plot no 23, Jote Joy Building, Rambhau Salgaonkar Marg, Cuffe Parade, Mumbai-400005, India

**Keywords:** Antiinflammatory activity, antioxidant activity, total polyphenol content, *Vitex negundo*

## Abstract

Reactive oxygen species are implicated in various inflammatory disorders. *Vitex negundo* is mentioned in Ayurveda as useful in treating arthritic disorders. The present work was undertaken to evaluate the antioxidant potential and anti-inflammatory activity of the plant. The total methanol extract of the plant was standardized in terms of total polyphenols. The standardized extract in a dose of 100 mg/kg caused a comparable reduction in edema with that of diclofenac sodium (25 mg/kg) when evaluated for antiinflammatory activity by carrageenan-induced rat paw edema method. The extract also exhibited a strong free radical scavenging activity by 1,1-diphenyl-2-picrylhydrazyl method and caused a significant reduction in the formation of thiobarbituric acid reacting substances when evaluated for its lipid peroxidation inhibitory activity. The results strongly suggest that radical quenching may be one of the mechanisms responsible for its antiinflammatory activity.

Ayurveda mentions several plants acting as antiinflammatory agents in arthritic disorders. *V. negundo* is a reputed drug in Ayurveda^1^ and is beneficial in inflammatory disorders. Normally about 5% of the inhaled oxygen is converted to harmful reactive oxygen species like O_2_^−^, H_2_O_2_, •OH^2^. In many inflammatory disorders there is excess activation of phagocytes and production of superoxide (O_2_^−^) radical^3^ which can harm surrounding tissue either by a powerful direct oxidizing action or indirectly as with hydrogen peroxide (H_2_O_2_) and hydroxyl radicals (•OH) formed from O_2_^−^ which initiate lipid peroxidation resulting in membrane destruction. The tissue damage then provokes inflammatory response by the production of mediators and chemotactic factors^4^. The reactive oxygen species are also known to activate matrix metalloproteinase (e.g. collagenase) causing increased destruction of tissues e.g. collagen damage seen in various arthritic disorders^5^.

Hence the agents that can scavenge these reactive oxygen species can be beneficial in the treatment of such inflammatory disorders. *V. negundo*, commonly known as *Nirgundi* is a widely available plant all over India. The easy availability of this plant, coupled with its reported usefulness in arthritic disorders prompted us to further explore its antiinflammatory and antioxidant activity.

The plant material was collected from Versova, Mumbai and plant sample was authenticated from Blatter Herbarium, St. Xavier's College, Mumbai. A herbarium specimen of this plant has been deposited at Principal K. M. Kundnani College of Pharmacy. Gum acacia, phosphoric acid, potassium ferricyanide, ferric chloride were of LR grade and obtained from local suppliers. Gallic acid and 1,1-diphenyl-2-picrylhydrazyl was procured from Sigma Aldrich Co. St. Louis, Mo. Ferrous sulphate, ascorbic acid, sodium chloride, potassium chloride, all of LR grade were obtained from local suppliers. Diclofenac sodium was obtained from Mac Labs, Mumbai as a gift sample.

The leaves were washed and dried in oven at a temperature not exceeding 50° and ground to obtain 40# powder. The powdered leaves were subjected to percolate extraction with 50% methanol. The extract was concentrated under vacuum. The total polyphenolic content of the whole percolate extract was determined using Prussian blue method^6^. The method involved the use of potassium ferricyanide and ferric chloride to obtain Prussian blue colour along with 1% gum acacia and 85% phosphoric acid as colour stabilizers. To 0.1 ml sample solutions, 1 ml of 0.016 M K_3_Fe(CN)_6_ was added followed immediately by 1 ml of 0.02 M FeCl_3_ in 0.1 N HCl. The content were mixed well and kept at room temperature for 15 min. The reaction mixture was stabilized by 5 ml of stabilizer containing water, 85% H_3_PO_4_ and 1% gum acacia in volume proportions of 3:1:1. The intensity of the blue colour was monitored at 700 nm by using Jasco V 550 UV/Vis spectrophotometer. The total polyphenols were estimated as gallic acid equivalents.

This method is based on reduction in absorbance of a methanol solution of coloured free radical, 1,1-diphynyl-2-picrylhydrazyl^7^. The test extract in various concentrations were added to 1,1-diphynyl-2-picrylhydrazyl solution (2 mg/ml) and the reduction in absorbance was monitored at 516 nm after 5 min. The activity was expressed as EC_50_ (the concentration of the test extract to cause 50% reduction in absorbance). The evaluation was carried out on Jasco V 550 UV/Vis spectrophotometer. Ascorbic acid was used as positive control. During the aerobic incubation of tissue homogenates, malondialdehydes are formed which on reaction with thiobarbituric acid produced a pink colour. The pink colour complex of thiobarbituric acid reacting substances was monitored at 532 nm^8^–^10^. Curcumin was used as positive control.

Mice were fasted overnight before the day of experiment and the liver was promptly excised after sacrificing the mice. The liver was weighed and perfused with 0.9% ice-cold saline. After washing, tissue homogenate was prepared in 10 volumes of ice-cold potassium chloride by homogenizing for 15 min.

The reaction mixture contained 0.2 ml of liver homogenates in a glass tube incubated with 0.1 ml KCl and 0.4 ml Tris-buffer (pH 7.5). To this solution, 20 μmol of adenine diphosphate was added followed by ascorbic acid (100 μmol). Extract (50 μl) was added in various concentrations followed by ferrous sulphate (10 μmol) and the reaction mixture incubated at 37° for 1 h. To this reaction mixture thiobarbituric acid was added and the mixture was boiled for 15 min. The tubes were then centrifuged at 4000 rpm for 10 min and cooled. Absorbance of the resulting pink chromogen was recorded at 532 nm.

The antiinflammatory activity was carried out on Wistar rats using the carrageenan-induced rat paw method^11^,^12^. Necessary permission was obtained from the Institutional Animal Ethics Committee. Rats of both sexes weighing between 150-250 g were randomly grouped in three groups as vehicle control (water+sodiumcarboxymethylcellulose), positive control (diclofenac sodium), test (extract). Suspension of diclofenac sodium (6.25 mg/ml) and extract was prepared in distilled water with sodium carboxymethylcellulose as suspending agent, carrageenan as 1% solution in normal saline was used as phlogistic agent.

Edema in one of the paws of the rat was induced by injecting into plantar aponeurosis 0.1 ml of 1% solution of carrageenan in normal saline. All vehicles, standard, and the test compound were fed to the rats one hour before the injection. Volume of the injected paw was measured plethysmographically immediately after the injection and then again at hourly intervals up to 4h. Edema was calculated for each interval for all groups and the mean of the edema at three-hour interval was calculated for test and standard and compared with control and % inhibition was calculated. Statistical analysis was carried out by applying student's t-test. P<0.05 was considered as statistically significant.

The whole percolate extract of *V. negundo* was found to contain predominantly polyphenols such as flavonoids as major constituents. Hence the extract was standardized in terms of total polyphenol content and found to contain 40.15% w/w of polyphenols as gallic acid equivalents. The phenolics, particularly polyphenols exhibit a wide variety of beneficial activities in mammals including antiviral, antibacterial, immune stimulating, antiallergic, antihypertensive, antiischemic, antiarrythmic, antithrombotic, hypocholesteromic, hepatoprotective, antiinflammatory, anticarcinogenic^13^. Flavonoids are an important group of polyphenols and are reported to inhibit prostaglandin synthesis, which are known mediators of inflammation^14^. The whole percolate extract of *V. negundo* when evaluated for its radical scavenging activity by 1,1-diphynyl-2-picrylhydrazyl method, exhibited a strong scavenging activity with EC_50_ value of 18.70 μg/ml against ascorbic acid as control which exhibited EC_50_ value of 2.85 μg/ml. When it was evaluated for its antioxidant activity by studying its lipid peroxidation inhibitory activity; it exhibited a concentration dependent inhibition of formation of thiobarbituric acid reacting substances (Table 1) with a potent antioxidant activity. Lipid peroxidation is responsible for damaging cell membranes thereby further intensifying inflammatory damage. A positive lipid peroxidation inhibitory activity of the extract of *V. negundo* could possibly be one of the reasons for its antiinflammatory activity. The extract in a dose of 100 mg/kg exhibited % inhibition in edema at the 3^rd^ h to the extent of 69.08% as against diclofenac sodium (dose, 25 mg/kg) which exhibited 67.16% reduction in edema (Table 2).

**TABLE 1:** LIPID PEROXIDATION INHIBITORY ACTIVITY

Material	Concentration (mg/ml)	% reduction in thiobarbituric acid reacting substances (TBARs)
*V. negundo* extract	1.87	14.87
(50% Methanol)	3.75	40.88
	5.62	53.89
	7.50	61.40
Curcumin	0.74	94.22
(Standard control)		

**TABLE 2:** ANTIINFLAMMATORY ACTIVITY: PERCENT EDEMA OVER TREATMENT PERIOD

Group	% Oedema ±SD			
	
	1^st^ h	2^nd^ h	3^rd^ h	4^th^ h
Vehicle control (n=14)	2.71±13.10	38.23±21.80	44.21±25.56	40.00±38.13
Diclofenac sodium (Standard control) 25 mg/kg, (n=13)	13.14±11.64	14.30±12.60	14.52±14.15*	10.82±10.98
*V. negundo* extract (50% Methanol) 100 mg/kg, (n=7)	7.18±3.39	13.83±10.16	13.67±5.14*	21.52±10.58

*p*-value:

* <0.05

The ethno medical use of *V. negundo* as a useful remedy in inflammatory and arthritic disorders could possibly be because of its excellent antiinflammatory and antioxidant potential. The present investigations have demonstrated a strong correlation between anti-inflammatory and antioxidant activities of *V. negundo*. The prevention of oxidative damage to tissue could therefore be one of the mechanisms responsible for the antiinflammatory effect shown by the plant. Confirmation of the antiinflammatory activity in animal models further justifies the traditional use of this plant for inflammatory disorders.
